# Anti-*Vibrio parahaemolyticus* compounds from *Streptomyces parvus* based on Pan-genome and subtractive proteomics

**DOI:** 10.3389/fmicb.2023.1218176

**Published:** 2023-07-06

**Authors:** Wenbin Liu, Peiyu Ou, Fangyuan Tian, Jingyang Liao, Yan Ma, Jie Wang, Xiaobao Jin

**Affiliations:** ^1^School of Basic Medical Sciences, Guangdong Pharmaceutical University, Guangzhou, China; ^2^Guangdong Provincial Key Laboratory of Pharmaceutical Bioactive Substances, Guangdong Pharmaceutical University, Guangzhou, China

**Keywords:** pan-genome, *Vibrio parahaemolyticus*, subtractive proteomics, *Streptomyces parvus*, bioflim

## Abstract

**Introduction:**

*Vibrio parahaemolyticus* is a foodborne pathogen commonly found in seafood, and drug resistance poses significant challenges to its control. This study aimed to identify novel drug targets for antibacterial drug discovery.

**Methods:**

To identify drug targets, we performed a pan-genome analysis on 58 strains of *V. parahaemolyticus* genomes to obtain core genes. Subsequently, subtractive proteomics and physiochemical checks were conducted on the core proteins to identify potential therapeutic targets. Molecular docking was then employed to screen for anti-*V. parahaemolyticus* compounds using a in-house compound library of *Streptomyces parvus*, chosen based on binding energy. The anti-*V. parahaemolyticus* efficacy of the identified compounds was further validated through a series of experimental tests.

**Results and Discussion:**

Pangenome analysis of 58 *V. parahaemolyticus* genomes revealed that there were 1,392 core genes. After Subtractive proteomics and physiochemical checks, Flagellar motor switch protein FliN was selected as a therapeutic target against *V. parahaemolyticus*. FliN was modeled and docked with *Streptomyces parvus* source compounds, and Actinomycin D was identified as a potential anti-*V. parahaemolyticus* agent with a strong binding energy. Experimental verification confirmed its effectiveness in killing *V. parahaemolyticus* and significantly inhibiting biofilm formation and motility. This study is the first to use pan-genome and subtractive proteomics to identify new antimicrobial targets for *V. parahaemolyticus* and to identify the anti-*V. parahaemolyticus* effect of Actinomycin D. These findings suggest potential avenues for the development of new antibacterial drugs to control *V. parahaemolyticus* infections.

## Introduction

*Vibrio parahaemolyticus* infections pose a significant global public health threat, with this bacterium ranking as the second most prevalent foodborne pathogen, second only to *Vibrio cholerae*. It is primarily present in seawater, seafloor sediments, and seafood products. The pathogen’s ability to infect a wide range of farmed species, including *Penaeus monodon* (Hossain et al., 2020), *Litopenaeus vannamei*, *Oreochromis niloticus* (Siddique et al., 2021), *zebrafish*, and *Procambarus clarkia* (Teng et al., 2022), can lead to substantial economic losses. Consumption of seafood contaminated with *Vibrio vulnificus* can result in adverse reactions, such as headaches, nausea, vomiting, diarrhea, and abdominal cramps in humans. In severe cases, the infection can progress to sepsis and even death (Raszl et al., 2016).

In response to the impact of *V. parahaemolyticus* on human health and aquaculture, the use of antibiotics has become increasingly widespread. However, this has led to the emergence of antibiotic-resistant strains of *V. parahaemolyticus*. Studies have demonstrated that *V. parahaemolyticus* has developed resistance to antibiotics such as ampicillin, sulfamethoxazole, cefazolin, and cefotaxime (Elmahdi et al., 2016). For instance, in Vietnamese seafood products, up to 85.71% of *V. parahaemolyticus* isolates showed resistance to at least one antibiotic (Vu et al., 2022). Similarly, in retail aquatic products in Nanjing, China, 71.6% of *V. parahaemolyticus* isolates were found to be multidrug-resistant (Zhou et al., 2022). Consequently, there is an urgent need to explore alternative strategies, such as finding new drug targets, to address the challenges posed by antibiotic resistance.

Drug targets serve as the cornerstone of new drug development. Screening large molecules as potential drug targets in the laboratory is both time-consuming and expensive. With the rapid development of sequencing technology, it has become more convenient to obtain genomic information of bacteria. The pan-genome, which encompasses all the genomes of different individuals of the same species, including the core genome and the accessory genome, represents a valuable resource for identifying drug targets and developing antibiotics. Specifically, core genes, which are present in all strains of the dataset, are suitable for drug target identification and antibiotic development. Recently, Dar HA et al. analyzed the pangenome of 150 *Mycobacterium tuberculosis* genomes and identified eight potential broad-spectrum drug targets. Furthermore, they discovered five potential drugs through molecular docking screening (Dar et al., 2020). Similarly, Kanwal Khan identified UDP-3-O-acyl-N-acetylglucosamine deacetylase as a potential pharmacological target for *Campylobacter ureolyticus* (Khan et al., 2022).

Essential genes and virulence factors play a crucial role in the discovery of antibacterial drugs. Essential genes are highly conserved across evolution and widely present in various disease strains, making them promising targets for treatment (Warrier et al., 2022). Lots of classical antibiotic have targeted essential bacterial macromolecular processes, encompassing nucleic acid, protein, and cell wall synthesis (Fields et al., 2017). In recent times, novel antibiotics with distinctive mechanisms of action, exemplified by SCH-79797 (Martin et al., 2020) and Darobactin (Imai et al., 2019), have emerged. These antibiotics specifically target essential bacterial functions: folate metabolism and membrane integrity, respectively, thereby achieving effective antimicrobial activity. Anti-virulence agents are considered to have a lower likelihood of developing resistance, reduce disease incidence, and exert no harmful effects on beneficial microbiota (Garland et al., 2017). Bacterial pathogens produce virulence factors, which represent potential targets for anti-virulence agents. These factors encompass adhesive fibers, mediators of biofilm formation, bacterial quorum sensing mechanisms, and specialized bacterial secretion systems. Numerous compounds have been designed to specifically target virulence factors in significant human bacterial pathogens. For instance, QseC inhibitors such as LED209 have demonstrated the ability to prevent *Vibrio parahaemolyticus*-induced acute hepatopancreatic necrosis disease (Yang et al., 2020). Furthermore, α-adrenergic blockers are being proposed as promising anti-virulence agents due to their capacity to hinder quorum sensing receptors and inhibit bacterial espionage (Almalki et al., 2022).

To tackle this challenge, the current study employed pan-genomic and subtractive genomic approaches to identify potential novel drug targets for *V. parahaemolyticus*. In addition, molecular docking techniques were employed to screen anti-*V. parahaemolyticus* compounds from *Streptomyces* secondary metabolites. Subsequently, the anti-*V. parahaemolyticus* activity of these compounds was validated experimentally.

## Materials and methods

### Collection of genomic data

A total of 58 whole genome sequences of *V. parahaemolyticus* were retrieved from the NCBI database^1^ on June 2nd, 2023. The detailed information regarding the genomes is presented in Supplementary Table S1, and the assembly levels of all these genomes are complete.

### Pangenome analysis of *Vibrio parahaemolyticus* strains

The Bacterial Pan-Genome Analysis tool (BPGA; Chaudhari et al., 2016) was utilized to perform pan-genome analysis on 58 *V. parahaemolyticus* genomes obtained from the NCBI database on December 9nd, 2022. The core genome of *V. parahaemolyticus* was constructed by applying a cut-off value of 50% sequence identity using USEARCH, and the resulting core genes were concatenated and aligned using MUSCLE to create a phylogenetic tree via the Neighbor-Joining method. The phylogenetic tree was visualized using Mega-X software. To conduct functional analysis of all the core, accessory, and unique genes, KEGG (Kyoto Encyclopedia of Genes and Genomes) was employed. Protein sequences associated with the core genome were used for subsequent drug target screening.

### Subtractive proteomics screening *Vibrio parahaemolyticus* drug targets

Ensuring the safety of patients’ medication is a prerequisite for drug treatment. Antibacterial drug targets that are homologous to human proteins may increase the risk of side effects. Essential genes are critical for the survival or normal function of an organism. Virulence factors refer to specific molecules or mechanisms that enable a pathogen (such as a virus, bacteria, fungus, or parasite) to cause disease in a host organism. Essential genes and virulence factors are the two main types of targets for the discovery of antibacterial drugs (De Backer and Van Dijck, 2003). Therefore, we conducted screening for non-human homologous proteins, essential proteins, and virulence factor proteins in the core genome to obtain potential drug targets.

The core genome was subjected to BLAST searches against the human genome (using an *E*-value >10^−3^), the DEG database of essential genes^2^ (using an *E*-value <10^−4^ and a bit score > 100; Luo et al., 2021), and the VFDB database of virulence factors^3^ (Chen et al., 2005). Drug targets were identified by taking the intersection of the BLAST results. The resulting proteins were further filtered based on their physicochemical properties, such as amino acid number, molecular weight, isoelectric point (pI), and grand average of hydropathicity (GRAVY) value, which were predicted using the Protparam tool.^4^ The subcellular localization of the proteins was predicted using CELLO^5^ (Novotný et al., 2022).

### molecular docking of putative drug targets with *Streptomyces parvus*’s secondary metabolites

The SWISS-MODEL software^6^ was used to predict the 3D structure of the target protein through homology modeling. The validity of the modeled protein was then assessed using the PDB SUM webtool.^7^

In our previous research, we found that a strain of Streptomyces WA5-1-29, which was isolated from the gut of *Periplaneta americana*, has significant anti-*V. parahaemolyticus* effects. However, the specific compound(s) responsible for the antibacterial activity remains unclear. The strain was further identified as *Streptomyces parvus* with 99.8% similarity to the strain *Streptomyces parvus* NBRC 112437 through the GenBank database (Supplementary Figure S1). We have developed an in-house compound library of *Streptomyces parvus* by utilizing the resources from the Streptomedb libraries^8^ and relevant literature (Moumbock et al., 2021). The compounds were chosen as ligands and subjected to docking with the target protein. Molecular docking studies were performed using Discovery Studio 2019 software, and protein binding sites were predicted using DeepSite^9^ (Jiménez et al., 2017).

### Fermentation, extraction, isolation, and spectroscopic analysis

In molecular docking studies, we found that Actinomycin D may be a potential anti-*V. parahaemolyticus* drug, so we performed a 40-liter scale fermentation of WA5-1-29 and extracted a crude sample using ethyl acetate. The crude sample was subjected to silica gel column chromatography (200–300 mesh) using a CHCl_3_/MeOH gradient (100,0, 50,1, 25:1, 15:1, 10:1, 5:1, 2:1, 1:1, and 0:100, v/v) to obtain 10 fractions (1–10). Fraction 1 was further purified using semi-preparative HPLC (YMC-Pack ODS-AQ chromatographic column, 250 × 10.0 mm, 5 μm) on a Waters 2,535–2,489 instrument and yielded 78.8 mg of an orange-red compound (80% MeOH/H_2_O). The compound was identified as Actinomycin D by analyzing its NMR spectra (acquired using a Bruker Avance III 600 NMR spectrometer) and MS data (measured on a Thermo Fisher TSQ Endura triple quadrupole mass spectrometer).

### Validation of the anti-*Vibrio parahaemolyticus* activity of Actinomycin D

1. The effect of Actinomycin D on *V. parahaemolyticus* growth

The determination of MIC and MBC was based on the broth micro-dilution techniques following CLSI (Clinical and Laboratory Standards Institute) guideline. The overnight culture of *V. parahaemolyticus* was diluted in Muller Hinton Broth (MHB) to a concentration of 1 × 10^5^ CFU/mL. Actinomycin D was also diluted in MHB to obtain concentrations ranging from 1.0 to 1024.0 μg/mL. In a 96-well plate, 100 μL of the *V. parahaemolyticus* solution and 100 μL of the Actinomycin D solution were added to each well, respectively. The plate was then cultured at 30°C for 24 h, followed by the addition of 10 μL of 0.5% TTC to each well. MIC was defined as the minimum Actinomycin D concentration that did not produce visible growth, while the minimum bactericidal concentration (MBC) referred to the lowest concentration of Actinomycin D that can kill 99.9% of bacteria in the culture medium. Each concentration was tested in 6 replicates.

Growth curve assay. The overnight culture of *V. parahaemolyticus* was diluted in nutrient broth supplemented with 3% NaCl to achieve a final concentration of 10^6^ CFU/mL. Actinomycin D was added to the bacterial suspension at varying concentrations of 16 μg/mL, 8 μg/mL, 4 μg/mL, 2 μg/mL, and 0 μg/mL, followed by incubation at 37°C for 24 h. The optical density at 600 nm (OD_600_) was measured at 3-h intervals. Each concentration was tested in 6 replicates.

1. Analysis of the effects of Actinomycin D on biofilm of *V. parahaemolyticus*

We analyzed the inhibitory and clearing effects of Actinomycin D on the biofilm of *V. parahaemolyticus* through two sets of experiments. In the inhibition experiment, *V. parahaemolyticus* cell suspensions (10^8^ cfu/mL) were inoculated into MHB containing Actinomycin D at concentrations of 1/8 MIC, 1/4 MIC, 1/2 MIC, and MIC. The 96-well plates were incubated at 30°C for 48 h. In the eradication experiment, after obtaining the mature biofilm using the same culture conditions as in the inhibition experiment, we carefully removed the MHB culture medium using a syringe. Next, we added 1/8 MIC, 1/4 MIC, 1/2 MIC, and MIC of Actinomycin D to the microtiter plate and incubated it at 30°C for 4 h.

Biofilm quantification was performed using the crystal violet staining method. First, we removed unattached planktonic bacteria from the 96-well plate by washing it with sterile PBS and dried it at 60°C for 30 min. The plate was then fixed with 95% methanol for 15 min, followed by staining with 0.1% crystal violet solution (500 μL per well) for 5 min. After washing the plate with PBS, we added 33% glacial acetic acid (200 μL per well) and incubated for 10 min to decolorize. The absorbance was measured at 595 nm using a microplate reader (Biotek, United States). The inhibition rate (%) was calculated using the formula: [(Control OD_595_ - Sample OD_595_)/Control OD_595_] × 100%.

Biofilm metabolic activity was determined using the XTT reduction assay. After washing the 96-well plate twice with PBS, 12.5 μL of XTT solution (1 mg/mL) and 1.0 μL of freshly prepared menadione solution (1 mmol/L) were added to each well. The plate was gently shaken and incubated in the dark at 37°C for 3 h. The absorbance was measured at 450 nm using a microplate reader (Biotek, United States).

Quantification of extracellular polysaccharides (EPS) was performed using the phenol-sulfuric acid method. The treated cells were harvested by centrifugation (4,000 rpm, 10 min, 4°C) and resuspended in PBS. The bacterial suspension was boiled for 10 min, cooled and 50 μL of Pronase E was added. The suspension was incubated at 37°C for 2 h. Next, 200 μL of 10% trichloroacetic acid (TCA) was added and incubated on ice for 30 min. The supernatant was collected by centrifugation (10,000 rpm, 30 min, 4°C), and an equal volume of ethanol was added. The mixture was incubated at −20°C for 1 h and centrifuged again to remove the supernatant (12,000 rpm, 20 min, 4°C). The pellet was treated with phenol (95%) and concentrated sulfuric acid and then boiled in a water bath for 10 min. The absorbance was measured at 595 nm using a microplate reader (Thermo, United States).

1. Analysis of Motility

The impact of Actinomycin D on the motility of *V. parahaemolyticus* was investigated through swimming and swarming exercise tests. For the swimming exercise test, 15 mL of TSB medium containing 0.3% (w/v) agar was prepared. In the swarming exercise test, 15 mL of TSB medium containing 0.5% (w/v) agar was used. Actinomycin D was added to the plates at final concentrations of 0, 1/8 MIC, 1/4 MIC, and 1/2 MIC. After a one-hour drying period, the center of each plate was inoculated with 5 μL of a *V. parahaemolyticus bacterial* solution (1 × 10^6^ CFU/mL), and the cultures were incubated at 37°C for 12 h (Liu and Wang, 2022). The motility diameter was measured using a ruler (mm), and pictures were captured using the Gel DOCTM XR + system (Bio-Rad). Each concentration was tested in three replicates.

1. CLSM Analysis

The survival of *V. parahaemolyticus* under Actinomycin D treatment was determined using a live/dead staining kit (Bestbio, Shanghai, China). Biofilms were allowed to form on glass slides placed in a 24-well microtitre plate, with and without Actinomycin D (1/2 MIC, 1/4 MIC, 0 μg/mL), and incubated for 24 h at 30°C. Subsequently, the glass slides were washed with 0.85% NaCl solution and stained with BBcellProbe® N01 and PI for 15 min in the dark. Finally, the slides were washed again with 0.85% NaCl s3X fluorescence confocal microscope.

### Statistical analysis

Each experiment was conducted a minimum of three times. The data are reported as mean values ± standard deviation. Statistical significance was determined using IBM SPSS v.19.0 software, employing Duncan’s multiple-range test and one-way ANOVA. A significance level of *p* < 0.05 was considered statistically significant.

## Results

### Pan and core genome analysis

**Figure 1:**
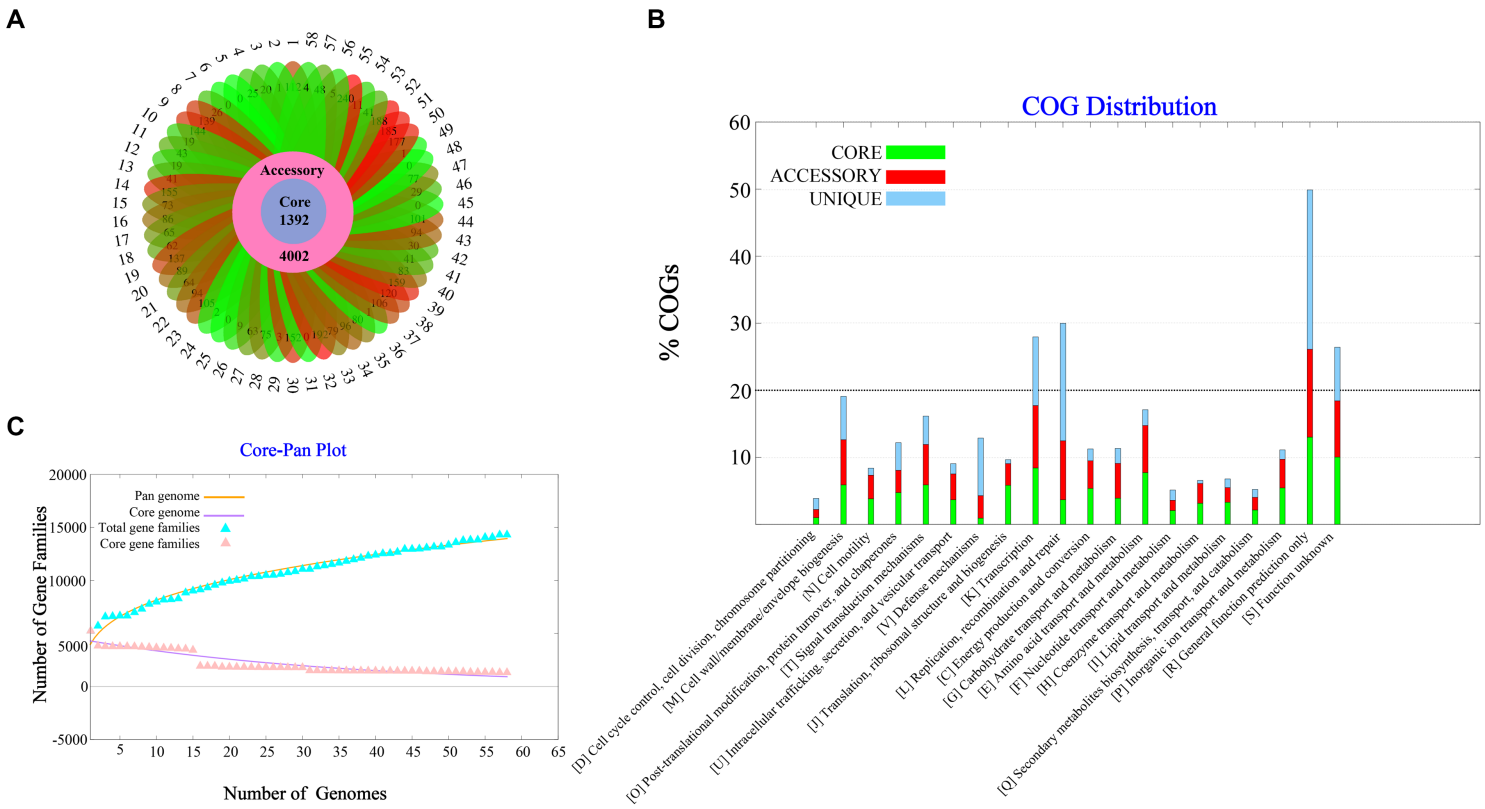
Pan-genome Analysis of 58 strains of *Vibrio parahaemolyticus* genome. **(A)** Core, accessory and unique gene families of 58 strains of *V. parahaemolyticus*. **(B)** A pan and core genome plot of *V. parahaemolyticus*. **(C)** COG distribution of core, accessory and unique genes.

A total of 58 complete *V. parahaemolyticus* genomes and related proteomes were downloaded from NCBI. Details of the strains such as Genome size, BioProject, GC%, Genes number, Host, are provided in Supplementary Table S1. Pangenome analysis of 58 *V. parahaemolyticus* genomes revealed that there were 14,379 gene families, out of which 1,392 were core genes (Figure 1A). Plotting of the gene families vs. genomes using power-fit curve equation: f(x) = 4031.59*x^0.3056^ and exponential curve equation: f1(x) = 4406.93*e^-x*0.02696^(Figure 1B). As a result, the pan-genome of *V. parahaemolyticus* was identified as almost OPEN (*b* value = 0.305638), global gene repertoire is likely to change in the near future. The strain 19-VB00998 had the highest number of accessory genes (*n* = 4,002), while the strain BTXS2 had the lowest number (*n* = 1,053). Six strains had no unique genes, while the strain VP157 had the most unique genes (*n* = 240) (Table 1). The COG functional annotation showed that the core genome was mainly enriched in genes conducting transcription, Amino acid transport and metabolism, Signal transduction mechanisms and Cell wall/membrane/envelope biogenesis. Accessory genome and unique genome were mainly enriched in genes conducting Transcription Replication, Recombination and repair, Cell wall/membrane/envelope biogenesis, Transcription (Figure 1C).

**Table 1:** Pan genome statistics of *Vibrio parahaemolyticus.*

Genome no.	Organism name	No. of core genes	No. of accessory genes	No. of unique genes	No. of exclusively absent genes
1	16-VB00198	1,392	3,816	112	1
2	17-VB00214	1,392	2,976	1	3
3	19-021-D1	1,392	3,542	20	0
4	19-VB00998	1,392	4,002	25	0
5	20-082A3	1,392	3,351	0	0
6	20-082E4	1,392	3,344	0	0
7	10,329	1,392	3,346	26	0
8	160,807	1,392	3,320	139	2
9	20130629002S01	1,392	3,255	144	0
10	20,140,624,012–1	1,392	3,286	19	9
11	20,140,722,001–1	1,392	3,498	43	2
12	20,140,829,008–1	1,392	3,410	19	7
13	20,151,116,002–3	1,392	3,353	41	14
14	20,160,303,005–1	1,392	3,684	155	1
15	BB22OP	1,392	2,861	73	44
16	BTXS2	1,392	1,053	86	970
17	CDC_K4557	1,392	3,009	65	0
18	CHN25	1,392	3,129	62	2
19	DHO76	1,392	2,891	137	48
20	DLM1799	1,392	2,859	89	0
21	DLM1805	1,392	2,830	64	3
22	FB-1	1,392	2,817	94	3
23	FDA_R31	1,392	3,064	105	8
24	FDAARGOS_51	1,392	3,266	2	1
25	FDAARGOS_191	1,392	3,317	0	0
26	FDAARGOS_662	1,392	3,252	9	0
27	FDAARGOS_667	1,392	2,993	63	2
28	FORC_006	1,392	2,900	75	2
29	FORC_008	1,392	2,946	3	0
30	FORC_014	1,392	3,035	152	0
31	FORC_018	1,392	2,369	0	247
32	FORC_022	1,392	3,063	192	0
33	FORC_023	1,392	2,755	79	4
34	FORC_071	1,392	2,988	96	3
35	FORC_072	1,392	3,003	80	7
36	LH24	1,392	3,142	1	0
37	LH80	1,392	3,041	106	3
38	LVP1	1,392	3,250	120	0
39	LVP2	1,392	3,303	159	3
40	LVP66	1,392	3,096	83	1
41	MAVP-Q	1,392	3,223	41	0
42	MAVP-R	1,392	3,339	30	1
43	MVP1	1,392	3,255	94	5
44	R13	1,392	3,729	101	0
45	R14	1,392	3,419	0	1
46	RIMD 2210633	1,392	3,336	29	0
47	S107-1	1,392	3,046	77	42
48	TJ-20	1,392	3,015	0	0
49	TJ-187	1,392	3,134	1	0
50	TJA114	1,392	3,134	177	6
51	UCM-V493	1,392	3,087	185	0
52	Vb0624	1,392	3,256	188	3
53	VP17	1,392	3,143	41	43
54	VP120	1,392	3,017	11	0
55	VP157	1,392	3,129	240	4
56	VPD14	1,392	3,312	5	0
57	XMM117	1,392	3,163	48	20
58	XMO116	1,392	3,130	4	0

### Subtractive proteomics revealed putative *Vibrio parahaemolyticus* drug targets

Subtractive proteomics was performed on the core genes. If a drug targets a human homologous protein, it may affect human metabolic function while producing biological activity; therefore, drug targets must not have homology to human proteins. A total of 1,100 proteins in the core proteins were identified as non-human homologous. Essential proteins are those required for a species to survive under any conditions. We retrieved 915 essential proteins of *V. parahaemolyticus* from the Database of Essential Genes. The core genes were then examined for virulence. Among these, 50 proteins were found to be virulent. Essential genes and virulence factors are the two main types of targets for the discovery of antibacterial drugs. Finally, we obtained 22 potential drug targets by taking the intersection of non-human homologous proteins, essential proteins, and virulence-associated proteins (Supplementary Figure S2). Detailed information on the drug targets is listed in Table 2.

**Table 2:** The physicochemical parameters of 22 potential drug targets.

Gene ID	Related genes	Number of amino acids	Molecular weight	Theoretical pI	Aliphatic index	GRAVY	Cellular localization
Gene1914	*epsL*	404	45273.37	5.14	94.31	−0.196	Unknown
Gene4291	*flhA*	699	75865.49	5.06	117.75	0.281	CytoplasmicMembrane
Gene2339	*flaD*	397	42310.86	4.97	82.39	−0.438	Extracellular
Gene2568	*fliO*	137	14714.45	9.85	122.48	0.307	CytoplasmicMembrane
Gene2523	*cheW*	164	18417.83	4.29	109.15	−0.061	Cytoplasmic
Gene2785	*flgJ*	308	34524.48	5.7	63.15	−0.736	Periplasmic
Gene4227	*cheR*	275	30822.76	9.31	92.22	−0.123	Cytoplasmic
Gene3839	*flhG*	295	32065.35	7.02	106.1	0.124	CytoplasmicMembrane
Gene2579	*cheA*	750	79593.49	4.59	99.27	−0.094	Cytoplasmic
Gene5160	*fliL*	167	17836.38	5.21	102.34	0.058	CytoplasmicMembrane
Gene4301	*fliJ*	147	17826.24	6.84	63.13	−1.088	Cytoplasmic
Gene4162	***fliN***	**136**	**14973.96**	**4.4**	**111.76**	**−0.218**	**CytoplasmicMembrane**
Gene4643	*lgtF*	264	30539.62	8.99	84.96	−0.431	Unknown
Gene60	*acpXL*	79	8779.74	3.87	111.01	−0.159	Cytoplasmic
Gene462	*vctD*	311	33664.33	8.87	134.41	1.012	CytoplasmicMembrane
Gene1683	*vctC*	251	27863.14	5.43	103.35	−0.016	CytoplasmicMembrane
Gene1799	*cysC1*	218	24385.56	5.16	91.61	−0.332	Cytoplasmic
Gene9778	*vscF*	82	9154.29	5.66	88.05	−0.538	Extracellular
Gene3168	*vscS*	88	9561.53	5.99	157.39	1.127	CytoplasmicMembrane
Gene1697	*vscG*	119	13038.86	4.69	93.78	−0.044	Unknown
Gene3569	*vscL*	212	23487.81	4.98	103.87	−0.169	Cytoplasmic
Gene1785	*virF*	286	32579.85	6.06	88.92	−0.33	Cytoplasmic

The bold values provided in Table 2 are used to highlight the selected target protein.

To identify potential drug targets, we further filtered the 22 candidates based on physicochemical parameters. Proteins with a length of fewer than 100 amino acids, high molecular weight, high pI, positive GRAVY value, low aliphatic index, and non-membrane localization were excluded from this study. After Subtractive proteomics and physiochemical checks, Flagellar motor switch protein FliN was selected as a therapeutic target against *V. parahaemolyticus*.

### Structure prediction and validation

**Figure 2:**
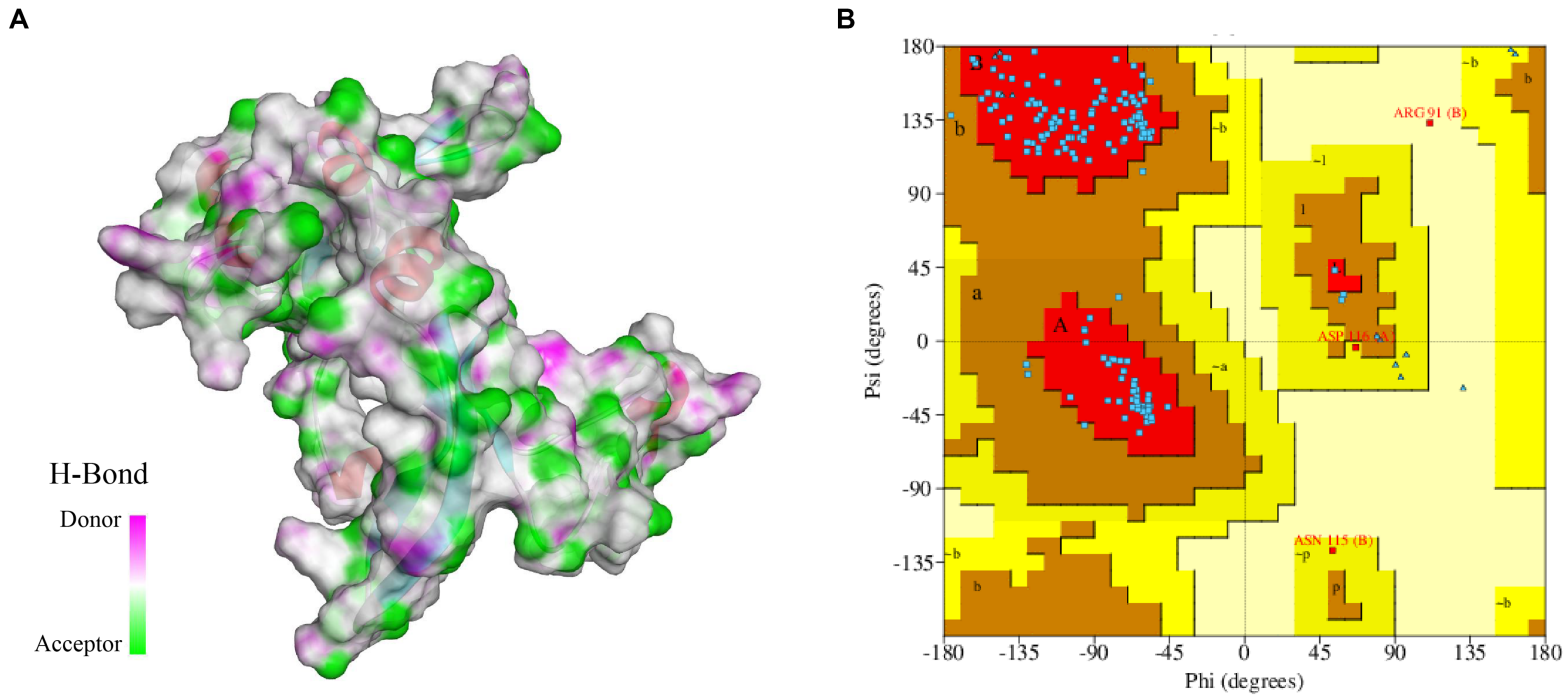
**(A)** 3D modeled structure of protein FliN (4612), the transparent surface of the protein is colored by hydrogen bond donor (pink) or acceptor (green). **(B)** Ramachandran Plot of protein FliN (4612). Red regions indicate residues in the most favored areas, brown regions represent residues in additional allowed areas, yellow regions denote residues in generously allowed areas, and white regions signify residues in disallowed areas.

In this study, we constructed a 3D structure model of FliN protein using homology modeling method. The structure of *Vibrio ichthyoenteri* ATCC 700023 FliN (PDB ID: F9RXD3.1.A) was chosen as a template due to its high sequence identity (91.91%) with the FliN protein of *V. parahaemolyticus*. The top modeled structure consisted of 1 sheet, 3 beta hairpins, 2 beta bulges, 4 strands, 2 helices, and 10 beta turns (Figure 2A). The Ramachandran plot analysis revealed that 93.28% of the model was located in the most favored region, indicating a high-quality structure. The top modeled structure was assessed with a QMEANDisCo Global score of 0.75 ± 0.07, and GMQE score of 0.82 (Figure 2B). These statistics demonstrate that the quality of the model was satisfactory for further analysis.

### Screening of compounds against targets

**Figure 3:**
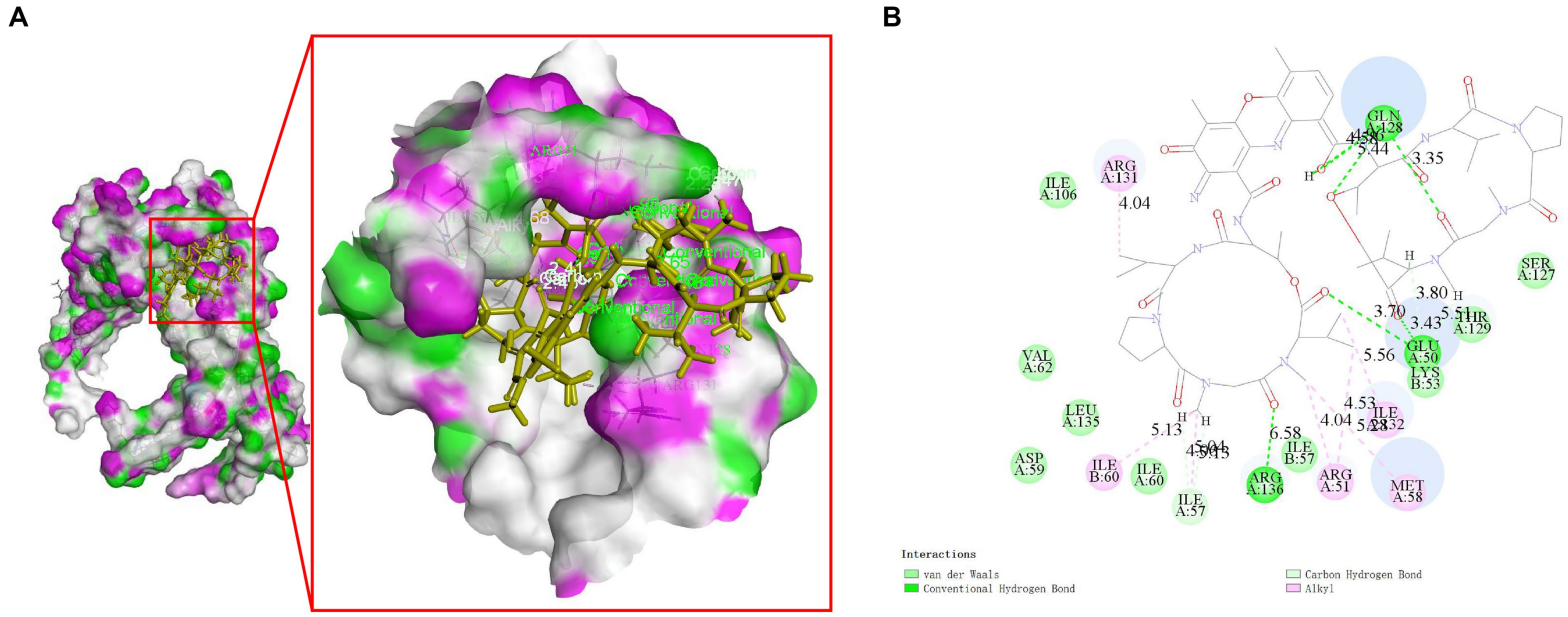
Molecular interaction between Actinomycin D with FliN (4612) in 3D **(A)** and 2D models **(B)**. Actinomycin D with FliN showing hydrogen bonding interaction with amino acid GLN128, GLU50, and ARG136. Hydrogen bonding interactions are shown as green dotted lines.

Molecular docking studies were conducted to investigate the potential of compounds from *Streptomyces parvus* to inhibit FliN. The Discovery Studio (LibDock) docking scores of ligands with drug targets are summarized in Supplementary Table S5. Actinomycin D exhibited strong binding energy with a score of 165.079. The 2D and 3D ligand-protein interaction analyses for Actinomycins D are depicted in Figure 3. The active sites of the receptors showed H-bonds and non-bonded interactions that stabilized the Actinomycin D interactions. Specifically, the oxygen atom of Actinomycin D formed six conventional hydrogen bonding interactions with Glu50, Gln128, Arg136 residues at distances of 3.70 Å, 5.44 Å, 4.58 Å, 3.35 Å and 6.58 Å, respectively. In addition, Actinomycin D formed six ALKYL interactions with Arg131, Ile60, Arg51, Met58 and Ile32. These findings suggest that Actinomycin D holds potential as an anti-*V. parahaemolyticus* drug.

**Figure 4:**
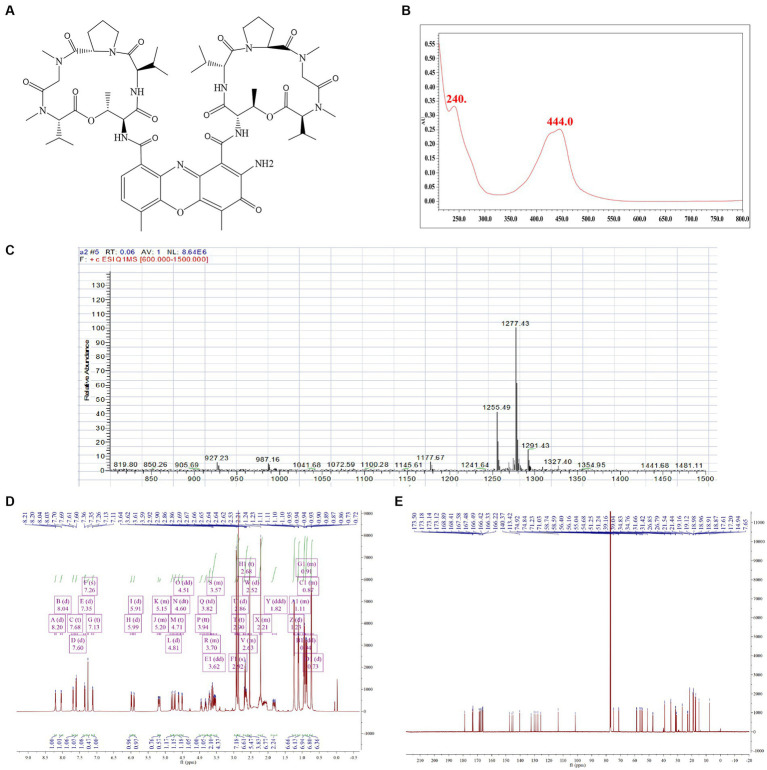
Structure identification of the purified compound. **(A)** Molecular structure of the Actinomycin D. **(B)** UV. **(C)** Mass spectrometry. **(D)** Proton (^1^H) nuclear magnetic resonance. **(E)** Carbon (^13^C) nuclear magnetic resonance.

To verify the biological activity of Actinomycin D (Figures 4A,B), we isolated 78.8 mg of red powder from the secondary metabolites of WA5-1-17. The molecular formula of the red powder was calculated as C_62_H_84_N_12_O_16_ based on the ESI-MS analysis at m/z 1255.49 [M + H]^+^ (Figure 4C) and ^13^C NMR data. The ^1^H and ^13^C NMR spectra of the red powder were closely similar to those of Actinomycin X_2_, differing in that the red powder has one less oxygen atom than Actinomycin X_2_. The ^13^C NMR spectrum exhibited that one signal is missing in the lower field, and one signal is added at δ 23. It is speculated that the carbonyl group in the proline moiety of the red powder is replaced by hydrogen (Figures 4D,E). Comparison of its MS and NMR data with those published in the literature enabled the identification of the red powder as Actinomycin D (Liu et al., 2019).

### Effect of Actinomycin D on *Vibrio parahaemolyticus* growth

**Figure 5:**
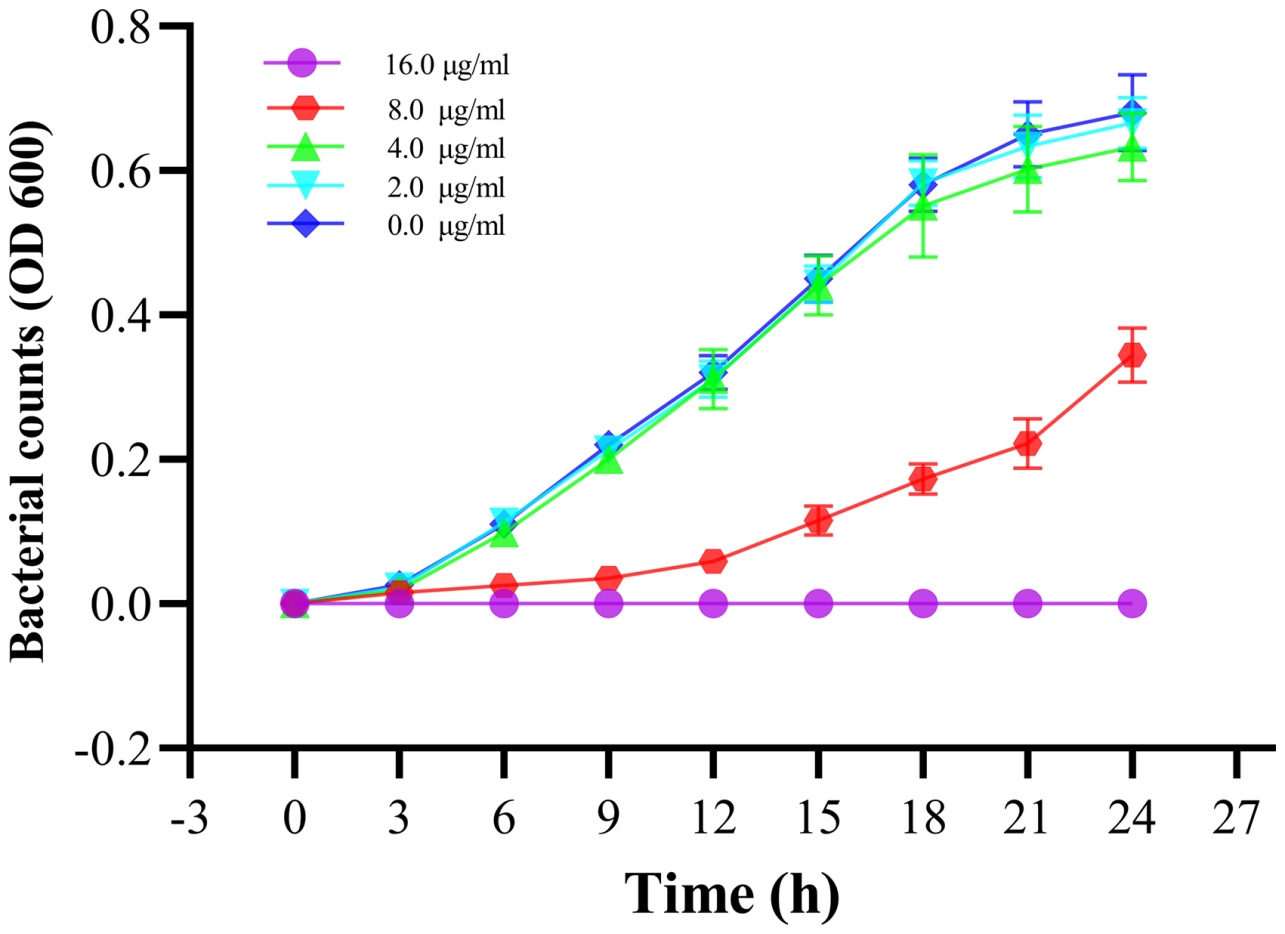
Effect of Actinomycin D on the growth curve of *V. parahaemolyticus*. Bars represent the SD. Each value represents the average of six independent measurements.

To verify the antibacterial effect of Actinomycin D on *V. parahaemolyticus*, we measured MIC and MBC using broth microdilution techniques. Increasing concentrations of actinomycin D significantly enhanced its antimicrobial activity against *V. parahaemolyticus*, with MIC and MBC values of 16.0 μg/mL and 64.0 μg/mL, respectively. The growth curve experiment provided further evidence, as it confirmed that Actinomycin D exerted a pronounced inhibitory effect on *V. parahaemolyticus* growth at a concentration of 16.0 μg/mL. Notably, when compared to the control group, Actinomycin D exhibited no significant impact on the growth curve of *V. parahaemolyticus* at concentrations below 8.0 μg/mL, as illustrated in Figure 5.

### Effect of Actinomycin D on *Vibrio parahaemolyticus* motility

**Figure 6:**
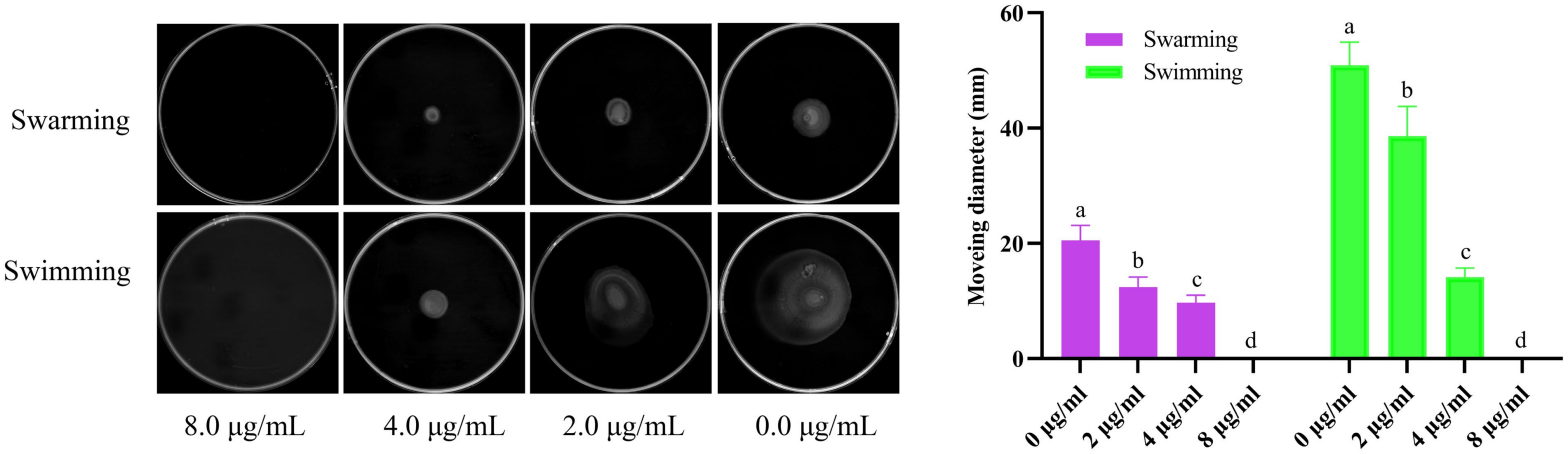
Effect of Actinomycin D on *V. parahaemolyticus* motility (*n* = 3). ^a-d^ Values with different letters differ significantly by Duncan’s multiple-range test (*p* < 0.05).

As shown in Figure 6, Actinomycin D inhibited both the *V. parahaemolyticus* swimming and swarming motility, and the motility diameter significantly reduced with the increase of Actinomycin D dose compared to that of the control (*p*<0.05). In contrast with the 0.0 μg/mL, 2.0 and 4.0 μg/mL of Actinomycin D had a significant reduction in the diameter of the swimming area by 24.2 and 72.2%, and it obviously reduced the swarming area by 39.7and 53.6%, respectively.

### Effects of Actinomycin D On biofilm formation of *Vibrio parahaemolyticus*

**Figure 7:**
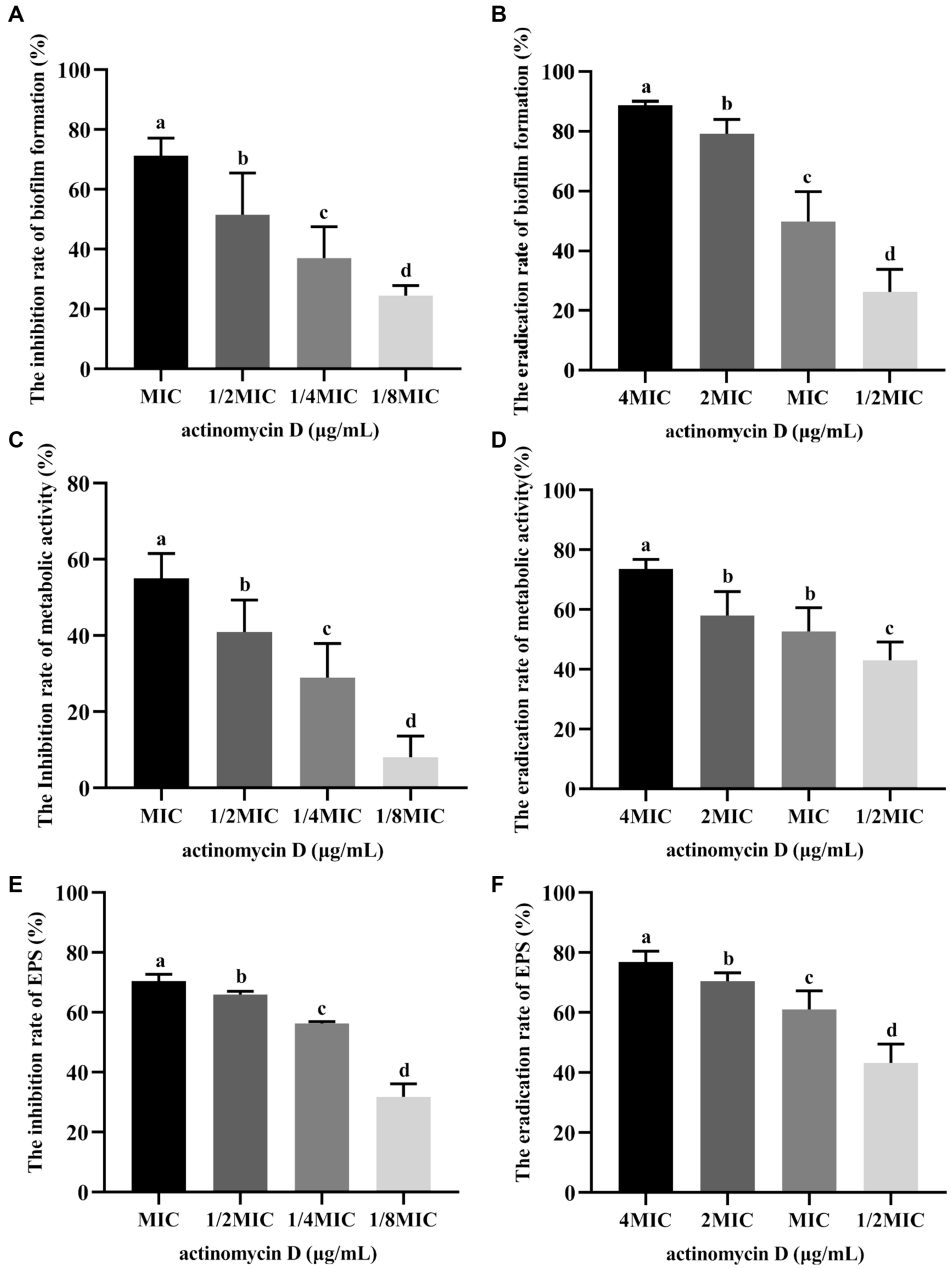
Effects of Actinomycin D on biofilm of *V. parahaemolyticus* (*n* = 6). **(A)** Inhibition effects on biofilm formation. **(B)** Eradicating effects on mature biofilms. **(C)** Reduction rate of metabolic activity on biofilm treated with Actinomycin D during biofilm formation. **(D)** Reduction rate of metabolic activity on mature biofilms treated with Actinomycin D. **(E)** Reduction rate of EPS on biofilm treated with Actinomycin D during biofilm formation. **(F)** Eradication rate of EPS on mature biofilms treated with Actinomycin D. The MIC of Actinomycin D against *V. parahaemolyticus* was 16.0 μg/mL. ^a-d^ Values with different letters differ significantly by Duncan’s multiple-range test (*p* < 0.05).

**Figure 8:**
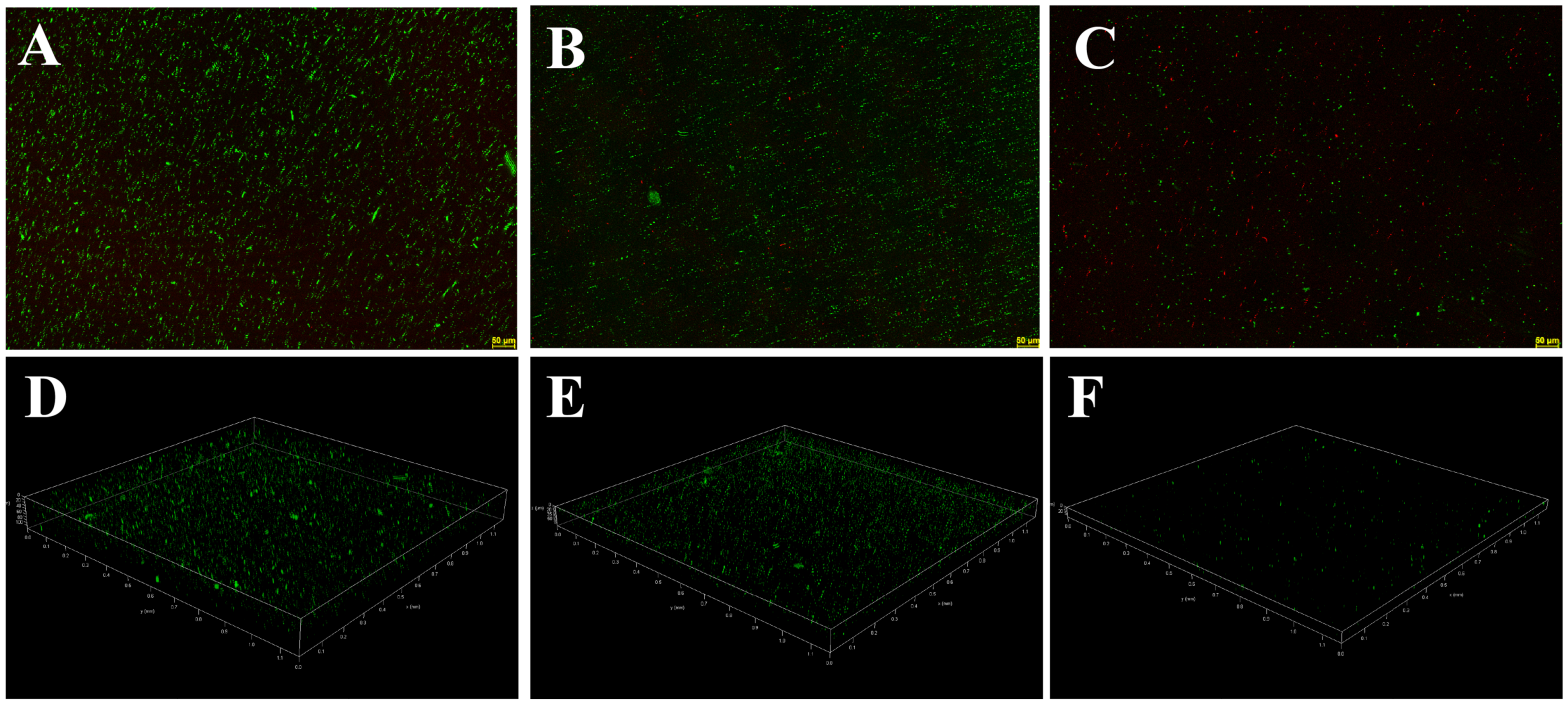
Confocal laser scanning micrographs of biofilms with different concentrations of Actinomycin D. **(A,D)** 0 μg/mL; **(B,E)** 4.0 μg/mL; **(C,F)** 8.0 μg/mL. Green represents viable bacteria and red for dead bacteria.

The inhibition and eradication effects of Actinomycin D on biofilms were determined using the crystal violet staining method. Biomass of *V. parahaemolyticus* biofilm decreased by 71.29 ± 5.31% and 51.56 ± 12.68% at MIC and 1/2MIC concentrations, respectively (Figure 7A). Eradication rates of Actinomycin D for mature biofilms were 88.73 ± 1.26% and 79.19 ± 11.97% at 4MIC and 2MIC concentrations, respectively (Figure 7B). Biofilms have been shown to alter bacterial metabolism in order to enhance their resistance to antibiotics. However, our XTT assay demonstrated that Actinomycin D was able to decrease the metabolic activity of the biofilms. The inhibition rate of Actinomycin D on metabolic activity ranged from 8.02 ± 5.15 to 55.00 ± 5.95% in the concentration range from 1/8MIC to MIC (Figure 7C). Eradication rates for mature biofilms were 73.63 ± 2.87% and 43.02 ± 5.54% at 4MIC and 1/2MIC, respectively (Figure 7D). Extracellular polysaccharides play a crucial role in biofilms, as they help bacteria firmly adhere to surfaces and promote bacterial aggregation, making them essential components of biofilms (Wang et al., 2022). Actinomycin D significantly reduced or eradicated EPS. At the MIC concentration, EPS inhibition rate was 70.43 ± 2.08% (Figure 7E), while for mature biofilms at 4MIC concentration, the eradication rate was 76.90 ± 3.22% (Figure 7F). We also utilized confocal laser scanning microscopy (CLSM) to investigate the impact of Actinomycin D on the formation of three-dimensional structures in *V. parahaemolyticus* biofilms, as illustrated in Figure 8. Comparing the treated biofilm with the untreated biofilm, it is evident that the untreated biofilm exhibited higher density and thickness (Figures 8A,D). Upon treatment with Actinomycin D, the density and thickness of the green fluorescence noticeably decreased, while the red fluorescence intensity increased proportionally with the dosage of Actinomycin D. These findings indicate a substantial reduction in viable bacteria and an increase in the number of dead cells.

## Discussion

One contributing factor to antibiotic resistance is the ongoing evolution of bacterial genomes. A critical feature of potential therapeutic targets is their prevalence among the majority of pathogenic strains within a species, coupled with their high degree of sequence conservation across different strains. In light of this, we conducted a comprehensive pan-genomic analysis of 58 *V. parahaemolyticus* genomes, focusing on the core genes present in all strains within our dataset for subsequent screening stages. As underscored in the introduction, identifying essential genes and virulence factors plays a pivotal role in the pursuit of novel antibacterial drugs. To ensure minimal disruption to human physiological functions, it is crucial that drug targets exhibit no homology with human proteins (Irfan et al., 2023). Therefore, we systematically screened for non-human homologous proteins, essential proteins, and virulence factor proteins within the core genome. We further filtered the candidates based on physicochemical parameters. Low molecular weight proteins (<110 kDa) are considered good drug targets as they are more accessible to drugs. Target proteins are more likely to be found in membranes (Bakheet and Doig, 2009). Mini-proteins (<100 amino acids) were considered insignificant (Bhattacharya et al., 2018). A negative GRAVY (grand average of hydropathicity) value of the protein indicates good hydrophilicity, while a high aliphatic index indicates good thermal stability.

After Subtractive proteomics and physiochemical checks, Flagellar motor switch protein FliN was selected as a therapeutic target against *V. parahaemolyticus*. The flagellar motor switch protein FliN is a major component of the flagellar motor switch complex, but its precise role remains unclear (Delalez et al., 2014). Flagellar-related proteins are promising targets for antibacterial drugs. According to research by Liang Yang et al., hydroxycoumarins may exert antibacterial effects by inhibiting the formation of *Ralstonia solanacearum* biofilms through the downregulation of flagellar genes fliA and flhC (Yang et al., 2016). Research by Sebastian Suerbaum et al. resulted in the development of a system for screening inhibitors of *H. pylori* flagellar biosynthesis and the discovery of a series of new antibacterial drugs (Suerbaum et al., 2022). Rongrong He et al. found that linalool emulsion could interact with the flagellar cap protein (FliD) and DNA of *Pseudomonas aeruginosa*, inhibiting biofilm formation and causing *P. aeruginosa* death (He et al., 2022). Since the virulence of *V. parahaemolyticus* is linked to its flagella, which also impacts the bacterium’s motility, FliN was chosen for further investigation in *V. parahaemolyticus*.

In the discovery of new antimicrobial agents, *in silico* approaches such as molecular docking are frequently employed due to their numerous advantages in terms of cost, time efficiency, and work effectiveness (Alharthi et al., 2023). Molecular docking encompasses both docking and reverse docking. Docking is commonly utilized to screen compounds in chemical libraries against specific protein targets, while reverse docking is typically employed to identify new targets for drugs(Wei et al., 2021, 2022). In a study conducted by Mahendra Kadiri et al., Penicillamine disulfide exhibited the highest binding energy with *Phytophthora infestans* target proteins, suggesting its potential as a novel biomolecule against late blight in potatoes (Kadiri et al., 2023). Through reverse molecular docking experiments, Raja Aadil Hussain Bhat discovered a novel antimicrobial peptide, KK16, which could bind to the omp protein and aerolysin protein, leading to cell lysis and reduced virulence (Bhat et al., 2022). In our study, we subjected an in-house compound library of *Streptomyces parvus* to *in silico* analysis against FliN. Actinomycin D exhibited a strong binding energy with a score of 165.079. A LibDockScore of ≥90 indicates a strong affinity between the ligand and receptor, facilitating ligand binding (Hu et al., 2022).

Actinomycin D is an antibiotic that is derived from *Streptomyces parvullus*. It exhibits activity against various tumors and has been clinically utilized to treat elapsed/refractory NPM1c-AMLs (Wu et al., 2021), Wilms’ tumor, pancreatic cancer, and osteosarcoma (Li et al., 2021), among others. In addition, Actinomycin D has antibacterial activity against several Gram-positive bacteria, including *Staphylococcus aureus* (Lin et al., 2021), *Staphylococcus epidermidis,* and MRSA (Xia et al., 2022), as well as Gram-negative bacteria, including *Pseudomonas aeruginosa* (Zeng et al., 2022) and *Xanthomonas oryzae* (Ogasawara et al., 2020), and fungi, including *Candida albicans*. There have been studies reporting that Actinomycin D can inhibit the formation of biofilms by *Staphylococcus epidermidis* (Lee et al., 2016), *Aspergillus fumigatus* (Seegers et al., 2023), and *Pseudomonas aeruginosa* (Zeng et al., 2022). However, currently there are no studies reporting on the effect of Actinomycin D on *V. parahaemolyticus* and its biofilm. Luckily, our molecular docking results indicate that Actinomycin D may be a potential anti-*V. parahaemolyticus* drug.

FliN, a component of the sodium-type switch complex, is known for its vital role in flagellar motility, torque generation, flagellar assembly, and control of flagellar rotation direction (McCarter, 2001). Our research unequivocally demonstrates the efficacy of Actinomycin D in impairing flagella-mediated motility in *V. parahaemolyticus*. The activation of motility-related genes sets off a cascade of events, triggering the expression and activation of various genes and regulatory systems associated with biofilm formation, including exopolysaccharide production, virulence properties, quorum sensing (QS), the type III secretion system (T3SS; Gu et al., 2020), and c-di-GMP signaling (Moisi et al., 2009). Targeting flagellar motility has emerged as a promising strategy for combatting *V. parahaemolyticus* infections. Previous investigations have highlighted the ability of certain compounds to reduce flagellar motility through mechanisms like transcription regulation, T3SS (Sun et al., 2019), and the QS signaling system (Satish et al., 2017), with minimal impact on bacterial growth. Diverging from these studies, our antimicrobial assessment clearly establishes Actinomycin D as a potent inhibitor of *V. parahaemolyticus*, as evidenced by its MIC and MBC values of 16.0 μg/mL and 64.0 μg/mL, respectively. Some studies have indicated that the compound, upon binding with flagella proteins, exhibits antibacterial, antimotility, and antibiofilm activities. For instance, silver ions and silver nanoparticles induce irreversible alterations in flagella structure, impairing movement and causing cell death (Li et al., 2017). In the case of *H. pylori*, β-lapachone exhibits remarkable bactericidal activity and anti-motility activity by specifically targeting the flagella (Suerbaum et al., 2022). Likewise, ferulic acid and p-coumaric display potent anti-*Salmonella Enteritidis* activity and significant antibiofilm effects, primarily due to their strong affinity for flagella proteins (Xu et al., 2022). Hence, taking into account our molecular docking results, we propose that Actinomycin D’s antibacterial and antibiofilm properties, as well as its antimotility effect, may be attributed to its direct binding to flagella proteins FliN.

## Data availability statement

The datasets presented in this study can be found in online repositories. The names of the repository/repositories and accession number(s) can be found in the article/Supplementary material.

## Author contributions

WL, XJ, and JW designed and planned the research. JL and FT finished pan-genome and subtractive proteomics, molecular docking studies. PO isolated and identified the bacterial strains. YM isolated and identified bioactive secondary metabolites. WL and XJ wrote the paper. All authors contributed to the article and approved the submitted version.

## Funding

This work was funded by the Guangzhou Science and technology planning project (202201010357), the Guangdong Natural Science Foundation (no. 2020A1515011097), the Public Welfare Research and Capacity Building Project of Guangdong Province (2017A020211008), and the Key Projects of Basic Research and Applied Basic Research of Guangdong Province Normal University (no. 2018KZDXM041).

## Conflict of interest

The authors declare that the research was conducted in the absence of any commercial or financial relationships that could be construed as a potential conflict of interest.

## Publisher’s note

All claims expressed in this article are solely those of the authors and do not necessarily represent those of their affiliated organizations, or those of the publisher, the editors and the reviewers. Any product that may be evaluated in this article, or claim that may be made by its manufacturer, is not guaranteed or endorsed by the publisher.

## Footnotes

^1^https://www.ncbi.nlm.nih.gov/genome/

^2^https://tubic.org/deg/public/index.php

^3^http://www.mgc.ac.cn/cgi-bin/VFs/v5/main.cgi

^4^https://web.expasy.org/protparam/

^5^http://cello.life.nctu.edu.tw/

^6^https://swissmodel.expasy.org/

^7^http://www.ebi.ac.uk/thornton-srv/databases/pdbsum/Generate.html

^8^http://132.230.56.4/streptomedb/

^9^https://www.playmolecule.com/
