# Supplementary material for: Anti-Vibrio parahaemolyticus compounds from Streptomyces parvus based on Pan-genome and subtractive proteomics
Source: Front Microbiol. 2023 Jul 6;14:1218176. doi: 10.3389/fmicb.2023.1218176 (PMC10361664; doi:10.3389/fmicb.2023.1218176)
Supplement: Supplementary file 1 [file Data_Sheet_1.zip › Supplementary material contents.DOCX]

Supplementary Material

Anti-*Vibrio parahaemolyticus* Compounds from *Streptomyces parvus* Based on Pan-genome and Subtractive Proteomics

Wenbin Liu^1,2^, Peiyu Ou^1,2^, Fangyuan Tian^1,2^, Jingyang Liao^1,2^, Yan Ma^1,2^, Jie Wang and Xiaobao Jin^1,2*^

*** Correspondence:** Xiaobao Jin^∗^: jinxf2001@163.com

# Supplementary Data

The amino acid sequence of FliN protein：

MEPSDDQKLADEWAAALGEDPSAPSIDVDEVLAAPLEELKDTSRPITDDERRKLDTIMDIPVTISMEVGRSQISIRNLLQLNQGSVVELDRLAGESLDVLVNGTLIAHGEVVVVNDKFGIRLTDVISQTERIKKLR

# Supplementary Tables

Table S1. Details information of all *Vibrio parahaemolyticus* strains.

Table S2. Essential genes of all *Vibrio parahaemolyticus* strains.

Table S3. VFDB genes of all *Vibrio parahaemolyticus* strains.

Table S4. Non-human homologous genes of all *Vibrio parahaemolyticus* strains.

Table S5. The LibDock score of ligands with FliN.

# Supplementary Figures
