# Supplementary figures and images for: Anti-Vibrio parahaemolyticus compounds from Streptomyces parvus based on Pan-genome and subtractive proteomics
Source: Front Microbiol. 2023 Jul 6;14:1218176. doi: 10.3389/fmicb.2023.1218176 (PMC10361664; doi:10.3389/fmicb.2023.1218176)

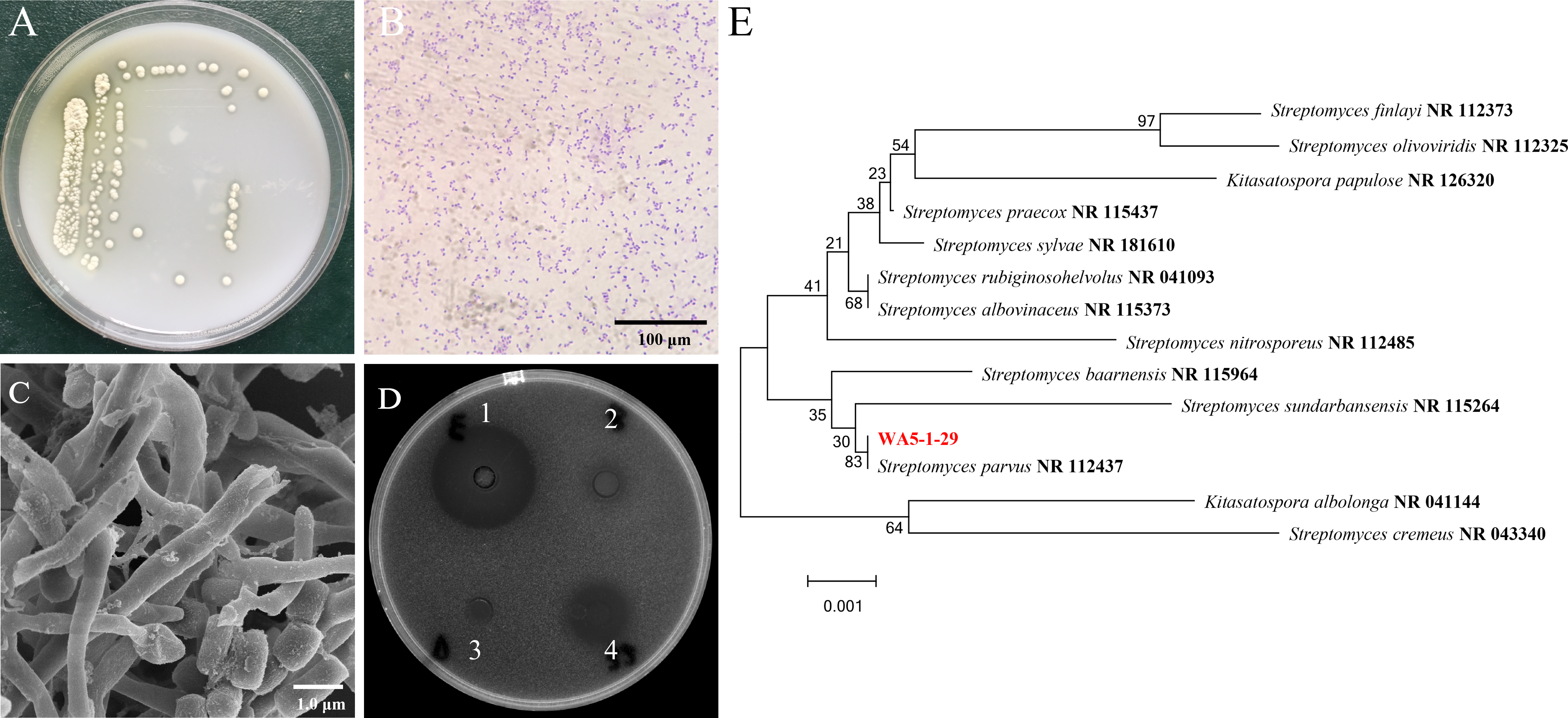

Supplement: Supplementary file 1 [file Data_Sheet_1.zip › Figure S1.TIF]

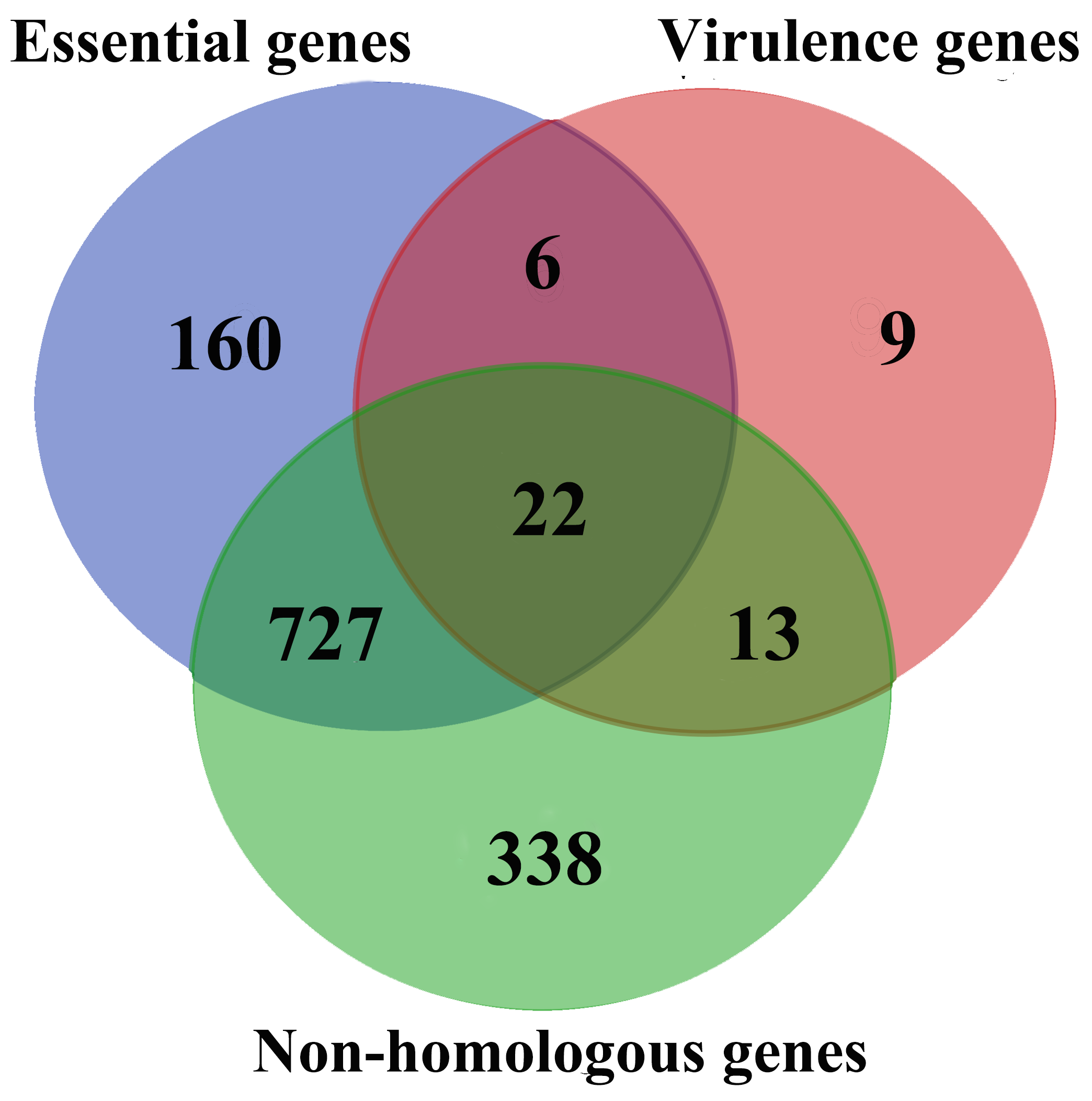

Supplement: Supplementary file 1 [file Data_Sheet_1.zip › Figure S2.TIF]
